# Transgenic Overexpression of Ephrin B1 in Bone Cells Promotes Bone Formation and an Anabolic Response to Mechanical Loading in Mice

**DOI:** 10.1371/journal.pone.0069051

**Published:** 2013-07-11

**Authors:** Shaohong Cheng, Chandrasekhar Kesavan, Subburaman Mohan, Xuezhong Qin, Catrina M. Alarcon, Jon Wergedal, Weirong Xing

**Affiliations:** 1 Musculoskeletal Disease Center, Jerry L Pettis VA Medical Center, Loma Linda, California, United States of America; 2 Department of Medicine, Loma Linda University, Loma Linda, California, United States of America; 3 Department of Biochemistry, Loma Linda University, Loma Linda, California, United States of America; 4 Department of Physiology, Loma Linda University, Loma Linda, California, United States of America; INSERM U1059/LBTO, Université Jean Monnet, France

## Abstract

To test if ephrin B1 overexpression enhances bone mass, we generated transgenic mice overexpressing ephrin B1 under the control of a 3.6 kb rat collagen 1A1 promoter (Col3.6-Tg^*efnb1*^). Col3.6-Tg^*efnb1*^ mice express 6-, 12- and 14-fold greater levels of full-length ephrin B1 protein in bone marrow stromal cells, calvarial osteoblasts, and osteoclasts, respectively. The long bones of both genders of Col3.6-Tg^*efnb1*^ mice have increased trabecular bone volume, trabecular number, and trabecular thickness and decreased trabecular separation. Enhanced bone formation and decreased bone resorption contributed to this increase in trabecular bone mass in Col3.6-Tg^*efnb1*^ mice. Consistent with these findings, our *in vitro* studies showed that overexpression of ephrin B1 increased osteoblast differentiation and mineralization, osterix and collagen 1A1 expression in bone marrow stromal cells. Interaction of ephrin B1 with soluble clustered EphB2-Fc decreased osteoclast precursor differentiation into multinucleated cells. Furthermore, we demonstrated that the mechanical loading-induced increase in EphB2 expression and newly formed bone were significantly greater in the Col3.6-Tg^*efnb1*^ mice than in WT littermate controls. Our findings that overexpression of ephrin B1 in bone cells enhances bone mass and promotes a skeletal anabolic response to mechanical loading suggest that manipulation of ephrin B1 actions in bone may provide a means to sensitize the skeleton to mechanical strain to stimulate new bone formation.

## Introduction

Osteoporosis is a common disease characterized by an age-dependent decrease in bone mineral density (BMD) and a microarchitectural deterioration of bone tissue with a consequent increase in the risk of developing fragility fractures of the hip, spine, and other skeletal sites [1]. The decrease in bone mass occurs when the body fails to form enough new bone to replace the amount of old bone resorbed leading to reduced bone strength. Therefore, studies on key regulatory molecules and their signaling pathways that control formation and activity of osteoblasts are essential to developing therapeutic strategies to identify novel anabolic therapies. In this regard, ephrin ligands and their receptors have been shown to play key roles in the growth and development of multiple tissues including the skeleton [2–5]. There are two types of ephrin ligands and their receptors. Ephrin As are membrane anchored proteins while ephrin Bs are transmembrane proteins. Ephrin B1 preferentially binds to EphB2 and B3 receptors with high affinity and interacts with EphB1, B4 and A4 receptors with low affinity [6,7]. It has been shown that both ephrin B1/2 and their receptors (EphB2, B3, B4, B6 and A4) are expressed in bone cells [8,9]. However, only ephrin B1 and 2 are expressed in osteoclasts during osteoclast precursor differentiation while ephrin B1/2 and their receptors are consistently co-expressed during osteoblast differentiation [8,9]. The interaction of ephrin B1/2 with their multiple receptors via cell-cell contact leads to the activation of a bidirectional signal in which both the receptor-mediated forward signal and the ligand-mediated reverse signal activate downstream signaling cascades [8,10]. Total disruption of the ephrin B1 gene in mice results in perinatal lethality and defects in skeletal patterning while mutations of the ephrin B1 gene in humans cause craniofrontonasal syndrome [3,10–12]. Mutation of the cytoplasmic tail of ephrin B1 that allows its extracellular domain to interact with ephrin receptors exhibits the same bone phenotypes as ephrin B1 knockout (KO) mice [2,10]. However, individual KOs of the EphB1, B2, B3 or A4 receptor, the major receptors for ephrin B1, showed mild behavior, nerve or digestive system phenotypes while KO of both EphB2 and EphB3 receptors resulted in embryonic lethality and a cleft palate phenotype [8,13–17]. Recently, Bush et al. found that ephrin B1 stimulated receptor forward signaling promoted mouse embryonic palate cell proliferation via activation of the ERK/MAPK signal transduction pathway, and also modulated EphB receptor expression through post transcriptional regulation [18]. Benson et al. demonstrated that ephrin B2 was upregulated at the sites of bone injury, and ephrin B2 stimulated forward signaling participated in fracture repair [9]. These experimental and genetic studies strongly suggested that ephrin B1/2 mediated reverse signaling via its cytoplasmic tail as well as its receptor-mediated forward signaling were essential in craniofacial development, bone formation and regeneration processes.

In our previous studies on the mechanisms by which locally applied mechanical strain exerts a bone anabolic response, we found that expression of EphB2 was stronglly induced in response to four point bending of the mouse tibia [19]. We also found that osteoblasts and bone marrow stromal (BMS) cells expressed both ephrin B1 ligand and EphB2 receptor and that interaction of ephrin B1 with EphB2 led to osteoblast differentiation and bone formation [5]. Because mechanical loading can stimulate EphB2 expression in bone cells, and because interaction of ephrin B1 ligand with the EphB2 receptor causes increased osteoblast differentiation, we hypothesized that mechanical loading on the bones of transgenic (Tg) mice that overexpress ephrin B1 can increase the interaction of ligand with native receptor by cell-cell contact *in vivo*, and induce bone formation. In this study, we generated Tg mice that express full length wild-type (WT) ephrin B1 under the control of the 3.6 kb rat collagen 1A1 promoter (Col3.6-Tg^*efnb1*^) and we evaluated the skeletal phenotype of Col3.6-Tg^*efnb1*^ mice. Furthermore, we detetrmined wheather the enhanced interaction of WT ephrin B1 with EphB2 with mechanical strain can induce new bone formation at the loaded site. Our studies demonstrate that Col3.6 promoter is active in both osteoblasts and osteoclasts, and that ephrin B1 Tg mice have both an increased bone formation and a skeletal anabolic response to mechaincal loading. Our findings suggest that manipulation of ephrin B1 actions in bone may provide a means to sensitize the skeleton to mechanical strain to stimulate new bone formation.

## Results

### Col3.6-Tg^*efnb1*^ mice express high levels of ephrin B1 in calvaria osteoblasts, bone marrow stromal cells and osteoclasts

**Figure 1:**
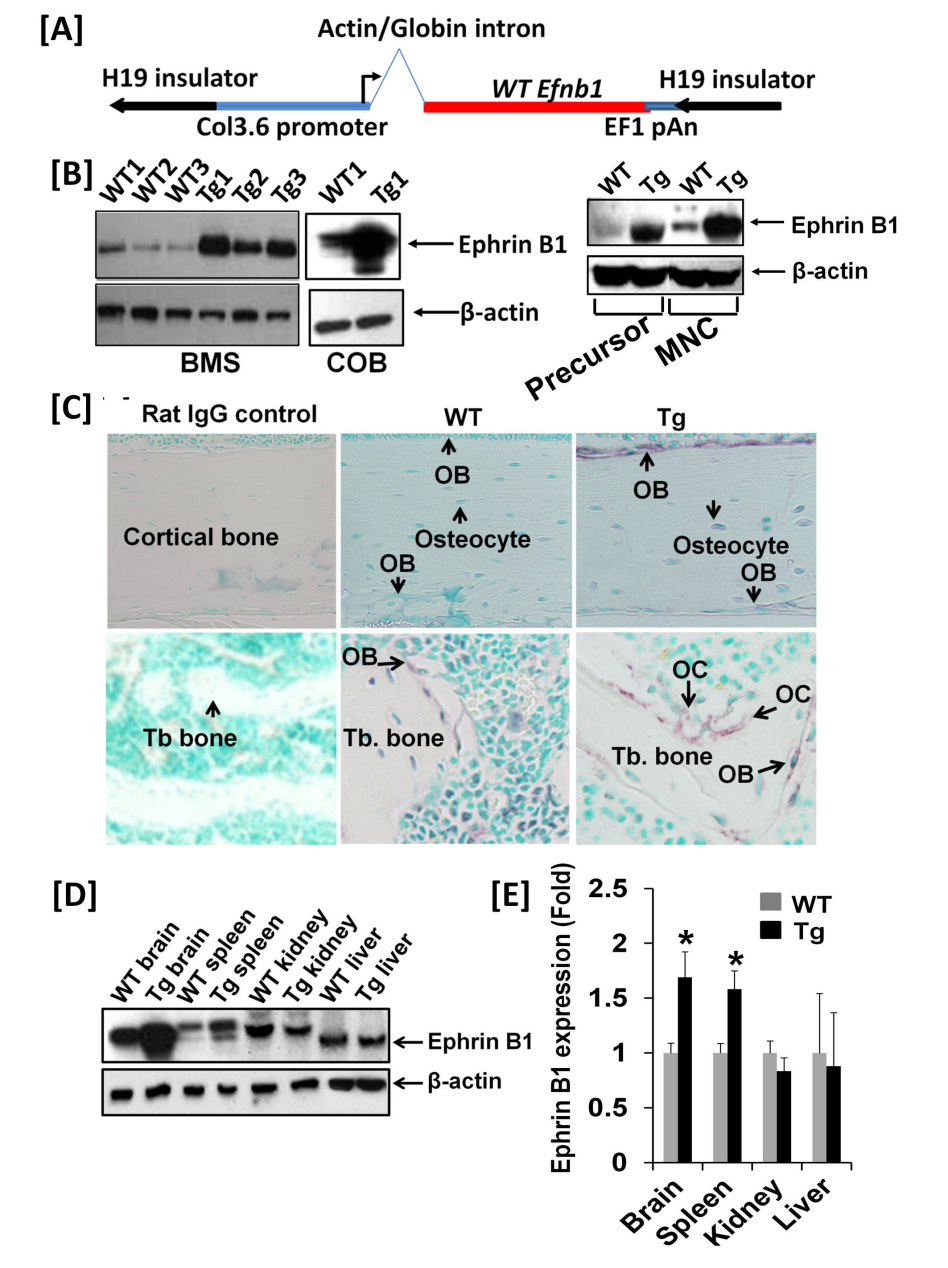
Col3.6-Tg^*efnb1*^ mice express high levels of ephrin B1 in the bone. **[A]** A schematic diagram of the transgene expression cassette. **[B]** Western blots show ephrin B1 overexpression in calvarial osteoblasts (COB), differentiated bone marrow stromal (BMS) cells and osteoclasts. BMS cells were differentiated in the presence of ascorbic acid and beta-glycerophosphate for 6 days prior harvesting for Western blot analyses. Osteoclast precursors derived from the spleen of WT and Tg mice were differentiated into multinucleated cells (MNCs) by 5-day treatment of M-SCF and RANKL. **[C]** Immunohistochemistry shows ephrin B1 overexpression in tibial periosteal, endosteal osteoblasts, osteocytes and trabecular surface osteobasts and osteoclasts. **[D]** A representative image of a Western blot shows ephrin B1 expression in the brain, spleen, kidney and liver in WT and TG mice. **[E]** Quantitative data of ephrin B1 expression from 3 independent experiments. Data are means ± SEM for N = 3. A star represents statistical significance (P < 0.05) when compared to WT littermate controls.

The expression of full length ephrin B1 in Tg mice was directed by a 3.6 kb rat Collagen 1A1 promoter and a chicken β-actin/rabbit β-globin chimeric intron (Figure 1A). The entire expression cassette also contained two mH19 insulators derived from the mouse *Igf2* gene on either side of the transcription unit. Both insulators were expected to protect transcriptional expression of the integrated *efnb1* gene from negative as well as positive influences from neighboring sequences at the integration site in an orientation independent manner. To confirm expression of transgene expression in Tg mice, we extracted the total cellular proteins from differentiated BMS cells and primary calvarial osteoblasts from 4 week-old mice for immunoblotting. Western blot analyses showed that ephrin B1 was highly expressed in differentiated BMS cells derived from three Tg mice, 12.4-fold higher in calvarial osteoblasts, and 6-fold higher in osteoclast precursors and multinucleated cells (MNCs) derived from Col3.6-Tg^*efnb1*^ mice as compared to the corresponding cells derived from WT control littermates (Figure 1B). Immunohistochemistry showed ephrin B1 overexpression in periosteal, endosteal and trabecular osteoblasts of the proximal metaphysis of the tibia of Col3.6-Tg^*efnb1*^ mice. Ephrin B1 remained overexpressed in osteocytes of the Tg mice (Figure 1C). Surprisingly, ephrin B1 was also expressed in active osteoclasts at the resorption lacunae of the trabecular bone. The ephrin B1 expression levels, as measured by Western blot analyses, were significantly higher in brain and spleen but not in kidneys and liver of the Tg mice when compared to WT littermate controls (Figure 1D & E).

### Gain of ephrin B1 function results in increased trabecular bone density and volume

**Figure 2:**
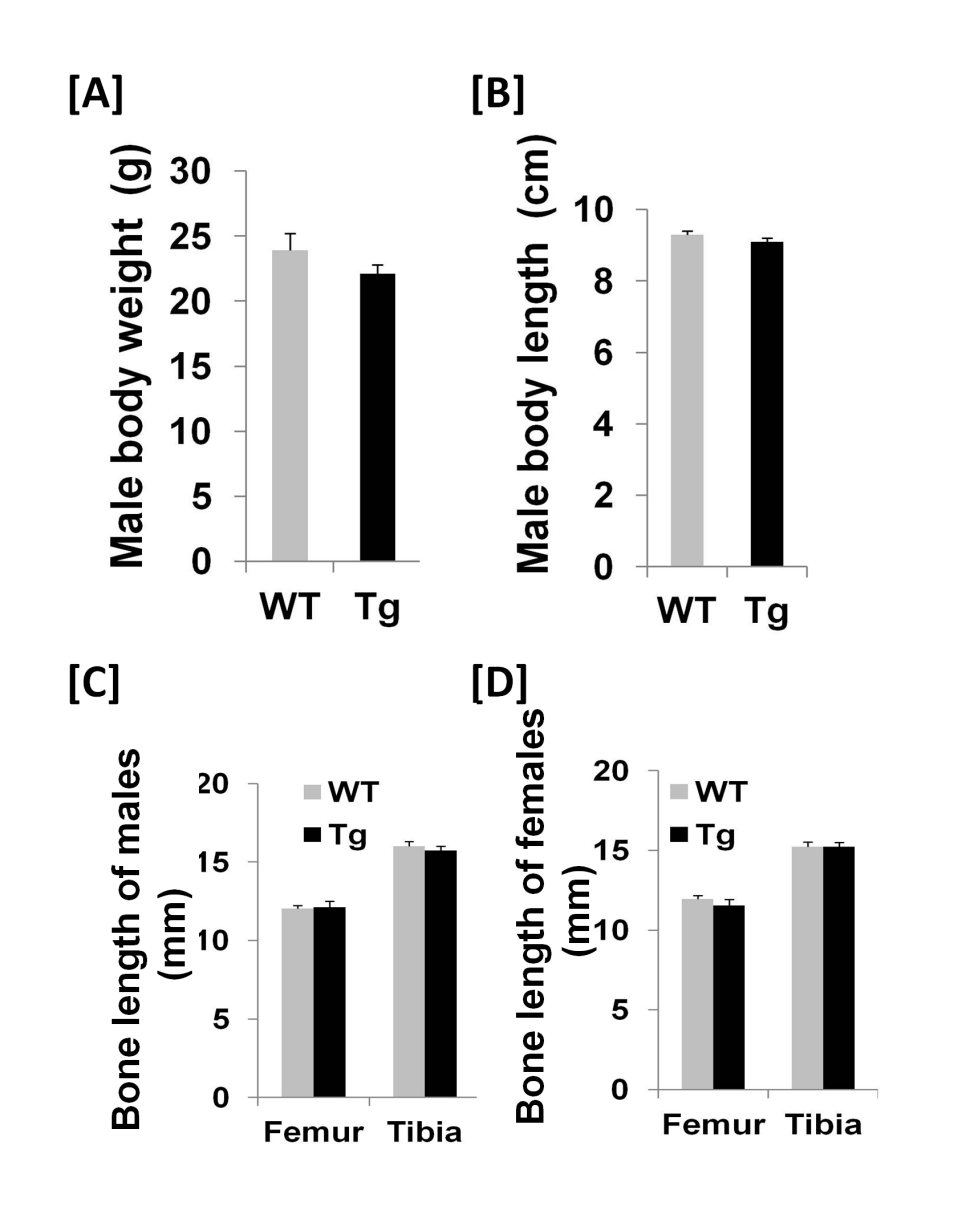
Overexpression of the ephrin B1 in bones does not affect body weight and bone size of the transgenic mice. Body weight **[A]** and body length **[B]** of 8-week old WT and transgenic (Tg) male mice were measured. Data are means ± SEM for N = 10. Bone length of 8-week old WT and Tg males **[C]** and females **[D]** were measured. Data are means ± SEM for N = 5 each.

**Figure 3:**
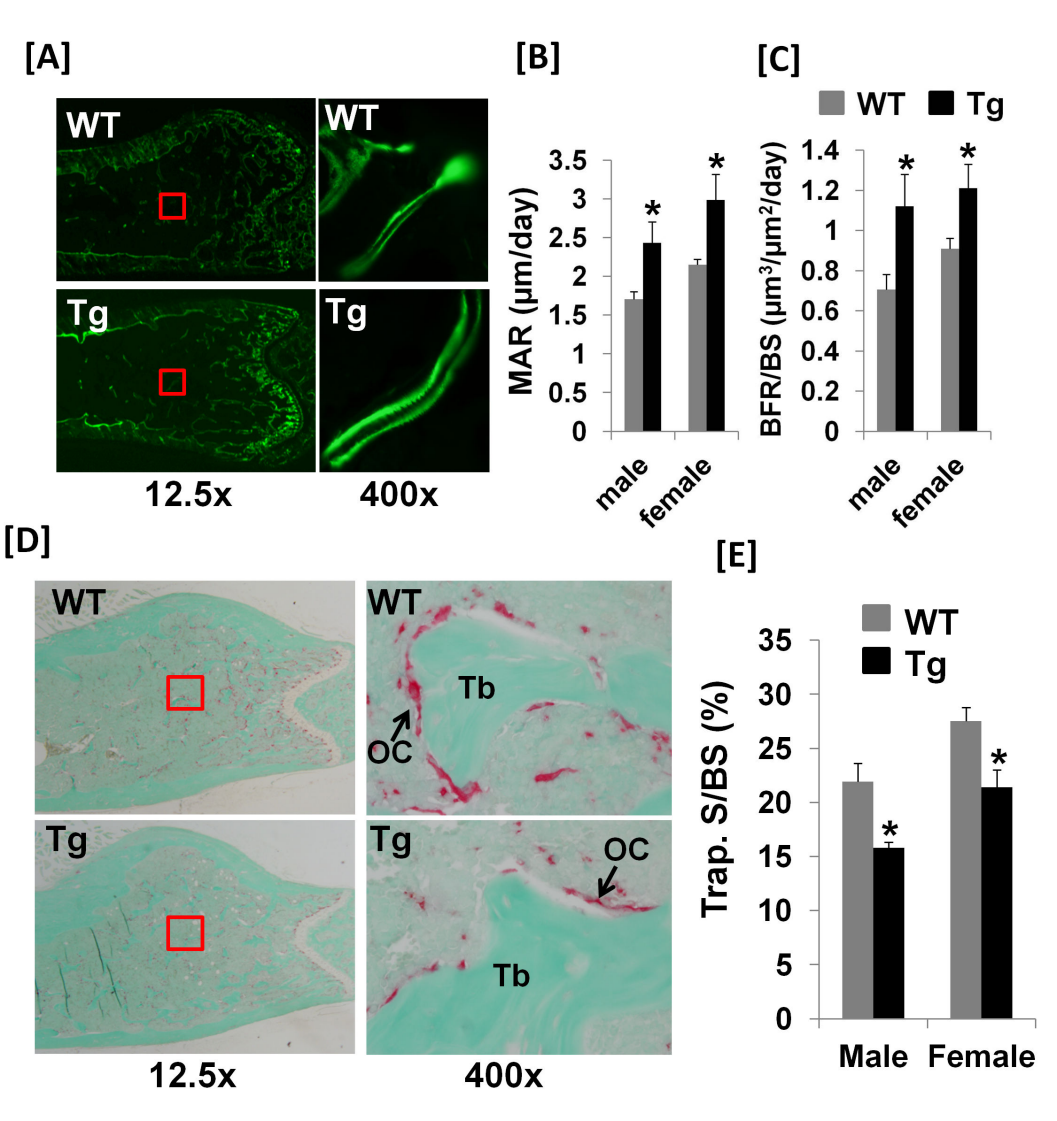
Col3.6-Tg^*efnb1*^ mice exhibit increased bone formation and reduced bone resorption. **[A]** Images of calcein double labeling of the distal metaphysic femurs of 10 week old WT and Tg mice. Mineral apposition rate (MAR) **[B]** and bone formation rate (BFR) **[C]** measured at the second spongiosa of the distal femural metaphysis of 10 week old WT and Tg mice. Data are means ± SEM for N = 7. **[D]** Images of TRAP staining of the distal femural metaphysis of 10 week old WT and Tg mice. **[E]** Quantitative data of the TRAP labeled surface to bone surface (Trap. S/BS) measured at the secondary spongiosia of the distal femural metaphysis of 10 week old mice. Data are means ± SEM for N = 8. A star represents statistical significance (P < 0.05) when compared to WT littermate controls.

Both body weight and body length of Col3.6-Tg^*efnb1*^ males were not significantly changed, neither were femur or tibia lengths of ephrin B1 Tg males and females as compared to corresponding 8 week old littermate control mice (Figure 2A & D). The body weight and length of Col3.6-Tg^*efnb1*^ females were not changed as well (data not shown). To determine the consequence of ephrin B1 overexpression under the control of the 3.6 kb rat collagen 1A1 promoter on bone formation and bone resorption processes, we performed histomorphometric studies at the femural metaphysis of 10-week old Tg and control mice. Figure 3A shows an increased width of newly formed bone between two calcein labels in Col3.6-Tg^*efnb1*^ mice as compared to control mice. The mineral apposition rate (MAR) was found to increase 44% and 39% in the Tg male and female mice, respectively (Figure 3B). The bone formation rate that was adjusted for differences in bone surface (BFR/BS) was increased by 58% and 33% at the femural metaphysis in Tg males and females, respectively (Figure 3C).

We next determined if overexpression of ephrin B1 in Tg^*efnb1*^ mice influenced bone resorption. Figure 3D & E show the data from tartrate-resistant acid phosphatase (TRAP) staining for trabecular surfaces examined at the distal metaphysis of the femurs of 10 week old mice. The percentage of TRAP labeled surface was reduced by 28% and 23% in the ephrin B1 Tg male and female mice, respectively, as compared to the gender-matched littermate controls (Figure 3E).

**Figure 4:**
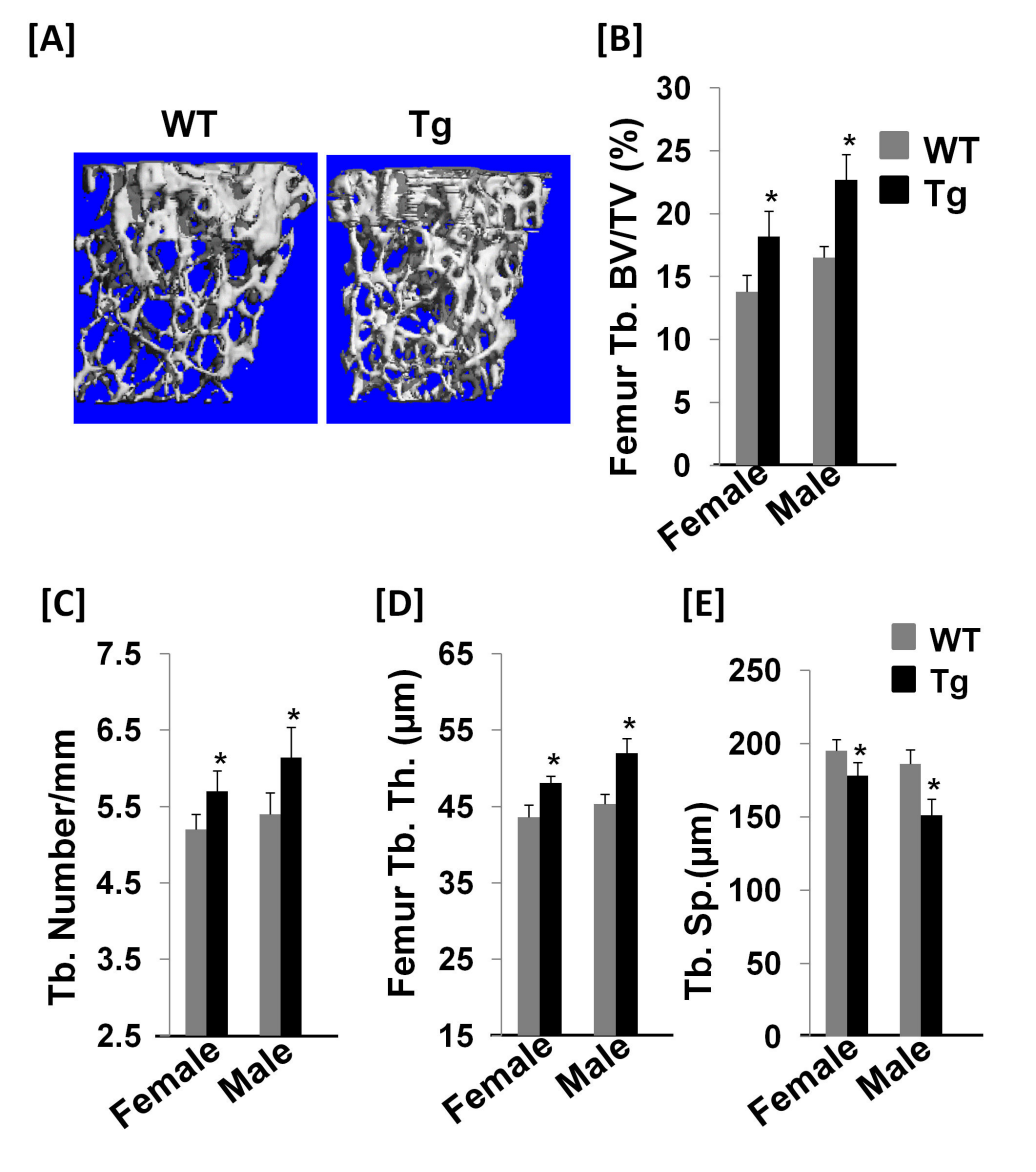
Col3.6-Tg^*efnb1*^ mice demonstrate increased trabecular bone and cortical thickness. **[A]** 3-D micro-CT images of the distal femural metaphysis of 8 week old WT and Tg male mice. [**B-E**] Quantitative data of μ-CT analyses, including trabecular bone volume to total volume (Tb. BV/TV), trabecular thickness (Tb. Th.), trabecular number (Tb. N), and trabecular spacing (Tb. Sp.). Data are means ± SEM for N = 7 per group. A star represents statistical significance (P < 0.05) when compared to WT littermate controls.

To further examine the 3-dimensional bone structure, the distal femurs of female and male Col3.6-Tg^*efnb1*^ mice as well as their littermate controls were used for µ-CT analyses. We found that the ratio of bone volume to total volume (BV/TV) was increased by 32 and 37% at the distal metaphysis of the femurs isolated from female and male Tg mice, respectively, as compared to the corresponding littermate controls (Figure 4A-B). Trabecular number was increased by 10% and 12% in the distal femurs of female and male Tg mice, respectively, while trabecular thickness was elevated by 10% and 14%, respectively (Figure 4C & D). Trabecular spacing was reduced by 8.5% and 11% in the distal femurs isolated from female and male Col3.6-Tg^*efnb1*^ mice, respectively, as compared to their corresponding littermate controls (Figure 4E).

### Overexpression of ephrin B1 stimulates osteoblast differentiation and inhibits osteoclast formation

**Figure 5:**
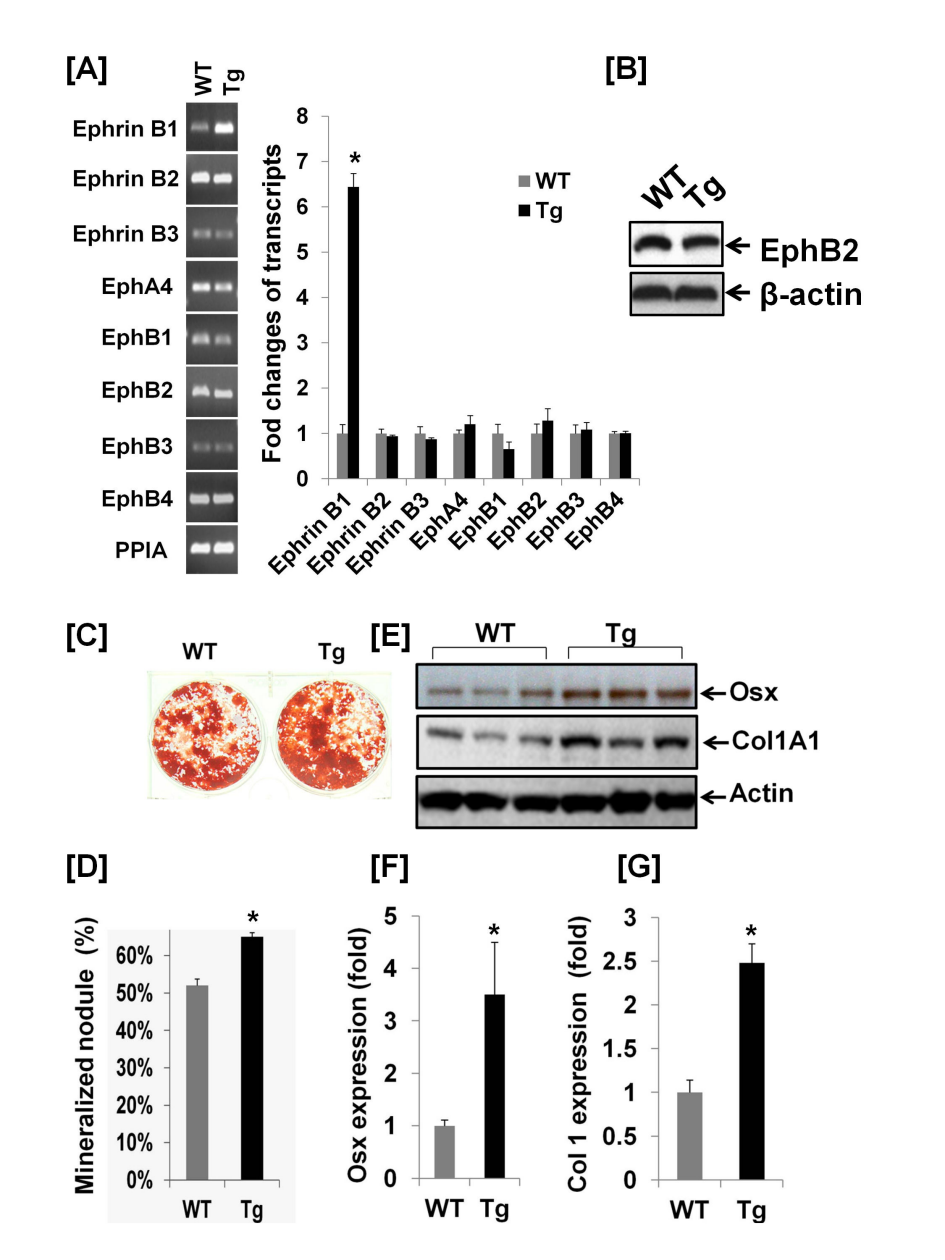
Overexpression of ephrin B1 increases osteoblast differentiation and osterix expression. **[A]** Multiple ephrin B ligands and ephrin receptors are expressed in bone marrow stromal (BMS) cells derived from WT and Tg mice, measured by real-time RT-PCR. **[B]** EphB2 expression in BMS cells, detected by Western blot analysis. **[C]** Images and quantitative data of mineralized nodules. **[D]** Quantitative data for mineralized nodule formation. BMS cells were differentiated for 24 days, followed by alizarin red staining and computerized quantification. Data are means ± SEM for N = 3 (3 mice/genotype). **[E]** Expression of osterix and collagen 1A1 (Col1) are increased in differentiated BMS cells, detected by Western blot analysis. BMS derived from WT and Tg mice were differentiated in the presence of ascorbic acid and beta-glycophosphate for 6 days prior to harvesting for Western blot analysis. [**F & G**] Quantitative data of osterix and col1 expression, measured by Western blot analysis. Data are means ± SEM for N = 4 (4 mice/genotype). A star represents statistical significance (P < 0.01) when compared to WT littermate controls.

Consistent with the Western blot data, real time PCR revealed a 6.5 fold increase in the expression of ephrin B1 transcripts in BMS cells of Col3.6-Tg^*efnb1*^ mice as compared to WT control mice (Figure 5A). Although the transcripts of other ephrin ligands and receptors including ephrin B2, ephrin B3 and EphA4, EphB1, EphB2, EphB3 and EphB4 were detected by real-time PCR, their expression levels were not significantly altered in BMS cells derived from ephrin B1 Tg mice compared to WT mice. EphB2 expression in BMS cells was further confirmed by immunoblotting with specific antibody against mouse EphB2 (Figure 5B). To examine the role of ephrin B1 overexpression in osteoblast differentiation, we cultured BMS cells derived from ephrin B1 Tg and WT mice and treated the cells with ascorbic acid for nodule formation assays. We found that the area of mineralized nodules was increased by 27% in BMS cells derived from Col3.6-Tg^*efnb1*^ mice as compared to control mice after 24 days of culture in mineralization medium (Figure 5C & D). To further determine the cause for increased osteoblastic function in the ephrin B1 Tg mice, we measured the expression levels of osterix, a transcription factor critical for osteoblast differentiation, and collagen 1A1, an early marker of osteoblast differentiation, after treatment of BMS cells from WT and Tg mice with ascorbic acid. We predicted that overexpression of ephrin B1 ligand would boost ephrin B1 reverse signaling in BMS cells derived from the Col3.6-Tg^*efnb1*^ mice by cell-cell contacts because BMS cells express endogenous EphB2 receptor to which ephrin B1 binds [5]. We found that osterix expression as determined by Western blot analyses using an osterix specific antibody was approximately 3.5-fold greater in Tg BMS cells that were incubated for 6 days in a mineralization medium containing ascorbic acid compared to WT BMS cells cultured under identical conditions (Figure 5E & F). Similarly, collagen 1A1 expression was increased by 2.5-fold in BMS cells derived from the Col3.6-Tg^*efnb1*^ mice as compared to the WT BMS cells cultured under the same culture conditions (Figure 5E & G). There was no change in Runx2 expression in the BMS cells derived from Tg mice as compared to the cells from WT control mice (data not shown).

**Figure 6:**
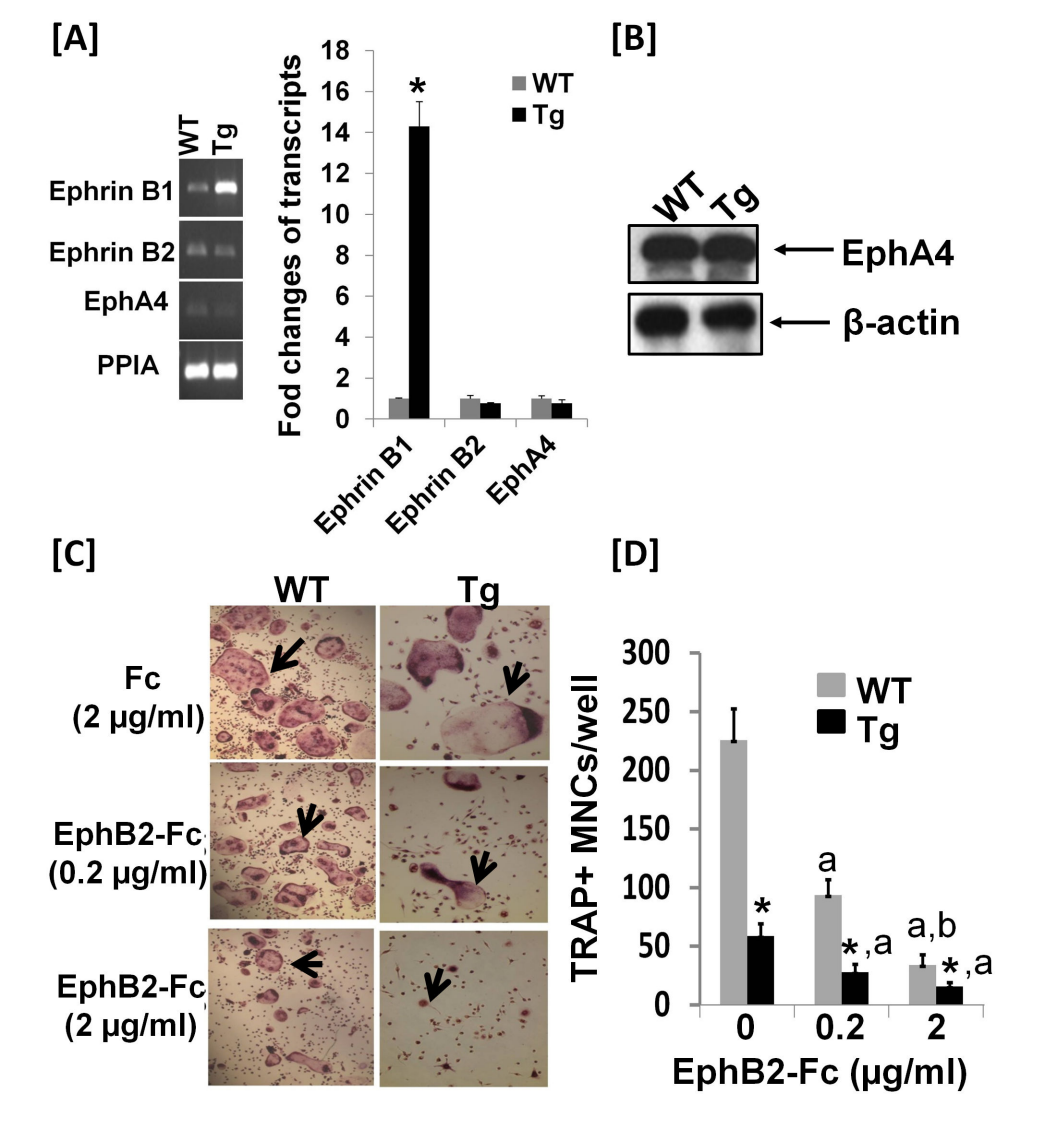
Overexpression of ephrin B1 in monocytes inhibits osteoclast differentiation. **[A]** Transcripts of ephrin B1, ephrin B2 and EphA4 in multinucleated cells (MNCs) derived from the spleen of WT and Tg mice detected by real-time PCR (N = 3 males). **[B]** Western blots show EphA4 expression in MNCs derived from one pair of WT and Tg male littermates. **[C]** Images of osteoclast differentiation. Monocytes derived from the spleen of WT and Tg mice were differentiated in the presence of M-CSF and RANKL, and treated with EphB2-Fc or control Fc for 8 days, followed by TRAP staining. The arrows indicate MNCs. **[D]** Quantitative data of MNC formation. Osteoclast precursors were pooled from 3 pairs of WT and Tg littermate males and seeded in 48-well plates in the presence of M-CSF and RANKL for differentiation. Data are the means ± SEM for N = 10 wells. Experiments were repeated for 3 times and similar results were obtained. A star represents statistical significance (P < 0.01) when compared to WT littermate controls. “a” indicates statistical significance (P < 0.01) when compared to vehicle control of the same cells. “b” indicates statistical significance (P < 0.01) when compared to 0.2 μg/ml treatment of the same cells.

To further determine if the 3.6 kb rat collagen 1A1 promoter directs ephrin B1 expression in osteoclasts, total RNA was extracted from mature multinucleated cells derived from the spleen of Col3.6-Tg^*efnb1*^ mice and WT control mice for real-time RT-PCR. As shown in Figure 6A, there was a 14-fold increase in ephrin B1 mRNA transcripts in mature osteoclasts derived from the spleen of ephrin B1 Tg mice as compared to the cells from WT mice. Transcripts of Ephrin B2 and EphA4 were also detected by real-time PCR, but the expression levels were not changed between the two cell genotypes. Expression of EphA4 protein in osteoclast precursors was further confirmed by Western blot analysis (Figure 6B). To determine if ephrin B1 overexpression in osteoclast precursors influenced the differentiation of precursors into mature MNCs, splenocytes derived from ephrin B1 Tg mice and WT control littermates were treated with soluble clustered recombinant EphB2-Fc protein that only contained the extracellular domain of the mouse EphB2 receptor, and can activate ephrin B1 reverse signaling but not EphB2 mediated forward signaling during M-CSF/RANKL-induced osteoclast differentiation. As shown in Figure 6C & D, eight days of M-CSF/RANKL treatment induced formation of mature MNCs from the precursors derived from the WT mice that was inhibited by exogenous addition of EphB2-Fc in a dose-dependent manner. Treatment of WT precursors with EpnB2-Fc at doses of 0.2 and 2 μg/ml inhibited MNC formation by 59% and 85%, respectively as compared with the cells treated with control Fc recombinant protein. Precursors derived from ephrin B1 Tg mice developed 76% fewer MNCs as compared to the cells isolated from WT control mice in the absence of clustered EphB2-Fc. Treatment of ephrin B1 Tg osteoclasts with 0.2 and 2 µg/ml clustered EphB2-Fc resulted in a greater inhibition of osteoclast formation. Formation of MNCs with at least 3 nuclei per cell was reduced by 82% and 58%, respectively, as compared to the corresponding WT osteoclasts under the same culture conditions.

### Overexpression of ephrin B1 in osteoblasts promotes a skeletal anabolic response to mechanical loading in Col3.6-Tg^*efnb1*^ mice

**Figure 7:**
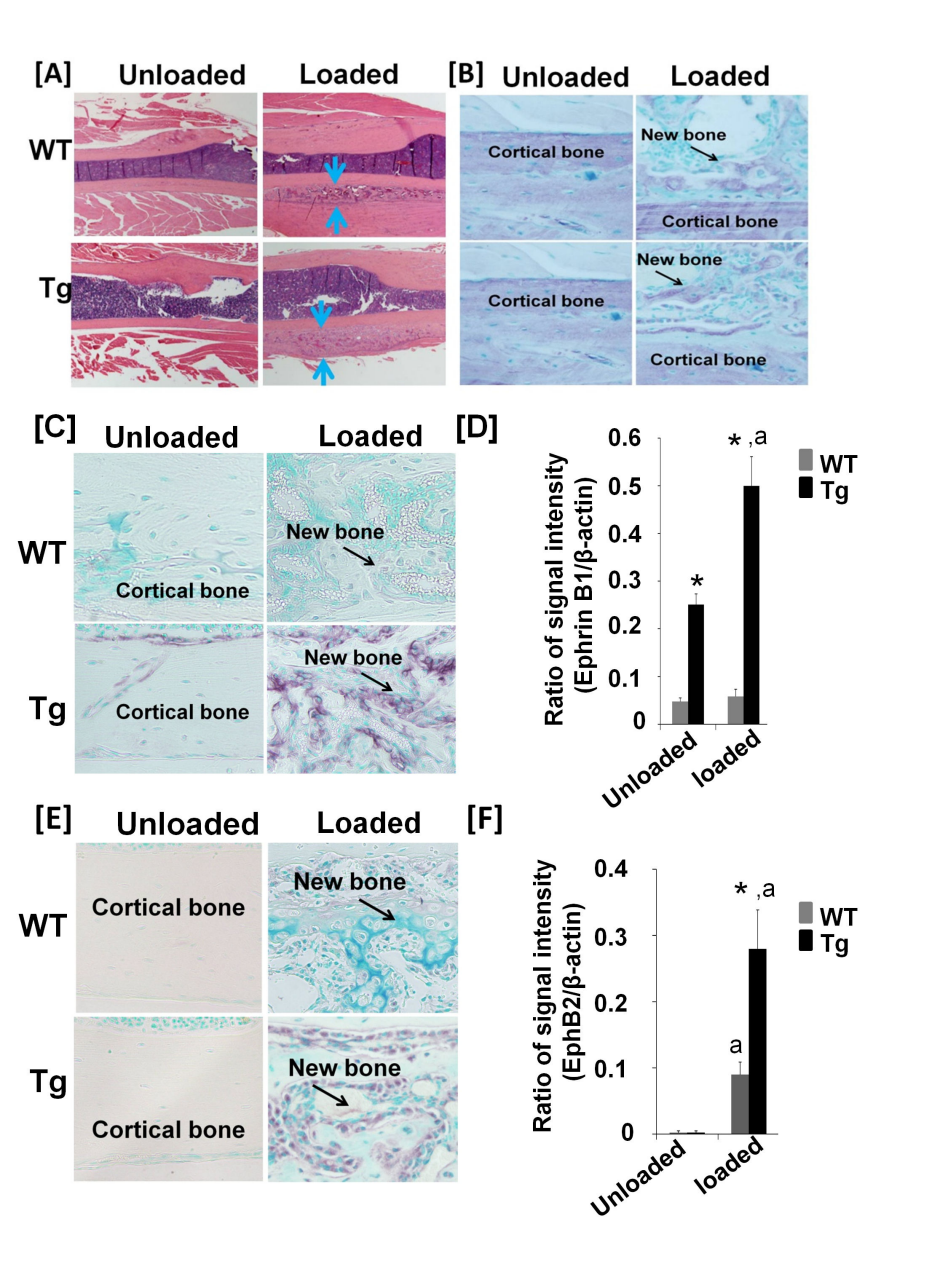
Mechanical loading stimulates bone formation and EphB2 and EphB4 expression in newly formed bone in transgenic mice. Tibia mid shafts from 12-week old WT and Tg, stained with H & E **[A]**. The right tibias of the 10-week old mice were loaded for 2 weeks, and the left tibias served as an unloaded control. Arrows indicate newly formed bone in response to mechanical loading. Expression of osteocalcin **[B]**, ephrin B1 **[C]**, and EphB2 **[E]** in newly formed bone are detected by immunohistochemistry. Quantitative data of ephrin B1 **[D]** and EphB2 **[F]** expression in newly formed bone, detected by immunohistochemistry are presented as the means ± SEM for N = 5 (3 pairs of littermate males and 2 pairs of littermate females). A star represents statistical significance (P < 0.01) when compared to WT littermate controls. “a” indicates statistical significance (P < 0.01) when compared to unloaded bones of the Tg mice.

**Figure 8:**
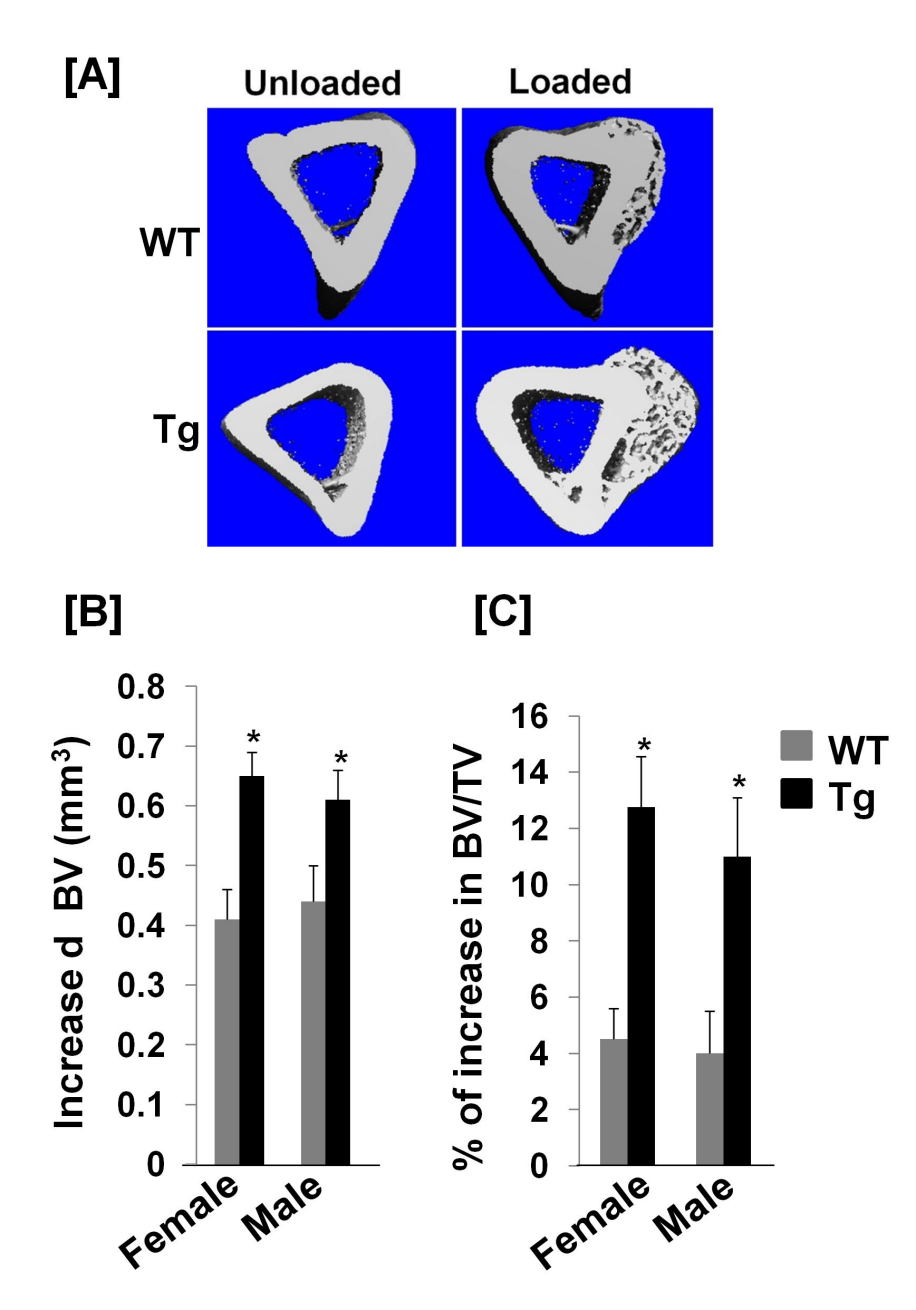
Overexpression of ephrin B1 in bone cells promotes a skeletal anabolic response to mechanical loading in transgenic mice. **[A]** Micro-CT images of the mid shaft of the tibias of12-week old WT and Tg male mice. [**B & C**] Quantitative data of increased total newly formed bone and BV/TV in response to mechanical loading. Data are the means ± SEM for N = 6 per group. A star represents statistical significance (P < 0.05) when compared to WT littermate controls.

Because mechanical stimulation can induce EphB2 expression in bone cells, and because interaction of ephrin B1 ligand with EphB2 receptor stimulates osteoblast differentiation [5,19], we next tested if ephrin B1 Tg mice would show an increased skeletal anabolic response to mechanical loading, by performing a 2-week four-point bending on the right tibias of 10 week old WT and Col3.6-Tg^*efnb1*^ mice. The left tibias of the same mice served as externally unloaded controls. The Col3.6-Tg^*efnb1*^ mice were intercrossed with C57BL/6J background mice for 4-5 generations. Figure 7A shows an increased thickness of newly formed periosteal woven bone in the Col3.6-Tg^*efnb1*^ mice as compared to WT control littermates. The periosteal bone cells from both WT and Col3.6-Tg^*efnb1*^ mice expressed high levels of osteocalcin, an osteoblast marker gene (Figure 7B). As expected, ephrin B1 was overexpressed in both loaded and unloaded bones of ephrin B1 Tg mice and its expressions were 5.3-fold and 9.1-fold higher in the unloaded tibia and the loaded tibia of the ephrin B1 Tg mice, respectively, than in corresponding tibias from WT control mice (Figure 7C & D). EphB2 expression was also induced in the tibias of WT mice in response to mechanical loading. Furthermore, its expression was increased by 3.2-fold in the loaded bone of Col3.6-Tg^*efnb1*^ mice compared to the loaded bones of WT control mice (Figure 7E & F). There was no detectable EphB4 expression in the newly formed bone of either WT or ephrin B1 Tg tibias (data not shown). With the increased expressions of ephrin B1 ligand and EphB2 receptor, the total volume of the right tibia (BV) in a 1.05 mm sampling site in Tg females and males was increased by 0.64 and 0.61 mm^3^, respectively, as compared to the corresponding region of the left tibia of the same mice while the total volume of the loaded tibia in WT control female and male mice was elevated by 0.41 and 0.44 mm^3^, respectively (Figure 8A & B). The relative cortical bone volume to total volume (BV/TV) was increased by 13% and 11% in the loaded region of the female and male Col3.6-Tg^*efnb1*^ mice, respectively, compared to unloaded Tg left tibias (Figure 8A & B). At the same experimental conditions, BV/TV was elevated by 4.5% and 4% in the loaded region of the female and male WT mice, respectively, as compared to unloaded WT left bones.

## Discussion

In this study, we generated Tg mice that overexpress full length mouse ephrin B1 under the control of the 3.6 kb rat collagen 1A1 promoter, and show that ephrin B1 was highly overexpressed in differentiated BMS cells, on osteoblast calvarial, endosteal and periosteal surfaces, in osteoclasts and in osteocytes of the long bones in Col3.6-Tg^*efnb1*^ mice. Characterization of the skeletal phenotype of Col3.6-Tg^*efnb1*^ mice show that overexpression of ephrin B1 in bone cells resulted in increased trabecular number, trabecular thickness, and trabecular bone volume with decreased trabecular separation. The elevated trabecular bone content in Col3.6-Tg^*efnb1*^ mice was caused in part by an increase in osteoblast differentiation and bone formation. Accordingly, *in vitro* studies show that interaction of ephrin B1 with clustered EphB2-Fc increased osteoblast differentiation, osterix and collagen 1A1 expressions in BMS cells. Furthermore, we demonstrate that the mechanical loading-induced increase in EphB2 expression and newly formed bone were significantly greater in Col3.6-Tg^*efnb1*^ mice than in WT littermates. Our findings suggest that manipulation of ephrin B1 actions in bone may provide a means to sensitize the skeleton to mechanical strain to promote new bone formation.

Boban et al. demonstrated that the 3.6 kb rat collagen 1A1 promoter is active in early mesenchymal progenitors, including preosteoblasts and osteoblasts, but that this promoter also contains regulatory elements that are active during osteoclastogenesis [20]. Scheller et al. also observed that Col3.6-Cre is widely expressed throughout the brain, centering near the distal third ventricle, third ventricle, and aqueduct [21]. Consistent with these findings, we found that the 3.6 kb rat collagen 1A1 promoter was also active in osteoclast precursors, in spleen, and in brain. In addition, our histomorphometric analyses demonstrated that the TRAP positive osteoclast surface to trabecular bone surface was significantly reduced, and that trabecular thickness was increased in Col3.6-Tg^*efnb1*^ mice. Consistent with reduced bone resorption in Col3.6-Tg^*efnb1*^ mice, osteoclast precursors derived from ephrin B1 Tg mice formed fewer TRAP positive osteoclasts as compared to the WT control cells after 8 days of RANKL/M-CSF treatment. Furthermore, treatment of osteoclast precursors derived from ephrin B1 Tg mice with clustered EphB2-Fc further inhibited osteoclast differentiation as compared to the same cells treated with Fc control protein. Together, these in vitro and *in vivo* data indicate that overexpression of ephrin B1 in osteoclasts also partially contributed to the increased trabecular and cortical bone volumes observed in Col3.6-Tg^*efnb1*^ mice by suppressing osteoclast formation and bone resorption. It remains to be determined if mouse *Igf2* insulators that we added to the 3.6 kb col1A1 promoter cassette partly contributed to the reduction of promoter specificity, and if overexpression of ephrin B1 in neurons contributed to the bone phenotype through a mechanism that involves direct sympathetic regulation of bone cells. Future studies will address these observations.

Previous studies have demonstrated that BMS cells and calvarial osteoblasts express both ephrin B1 and its cognate EphB2 receptor [5], and that osteoclasts and macrophages express ephrin B1, ephrin B2, EphA2, and EphA4 [5,22,23]. Ephrin B1 binds to EphB1, B2, B3, B4 and A4 receptors [6–9,18]. In the cells that co-express both ephrin ligands and receptors, the ephrin ligand and receptor proteins can be segregated into distinct membrane domains from which they signal biological effects via cell surface interactions [24]. Thus, both forward and reverse signaling is feasible upon contact of osteoclast to osteoblast, osteoblast to mesenchymal stem cell or osteoblast to osteoblast on the bone surface. In this regard, recent studies found that interaction of ephrin B2 with EphA2 in osteoclasts activates both forward and reverse signaling leading to osteoclast differentiation [25], and ephrin B1 reverse signaling induces osteoblast differentiation [5]. Interaction of ephrin B2 with EphB4 in the osteoblast lineage is required for late stage osteoblast differentiation, and this interaction controls osteoblast support for osteoclast formation via limiting RANKL production [26]. On the other hand, it has been reported that ephrin B1-initiated forward signaling of EphA4 promotes cortical cell division in the brain [7], and the disruption of the EphA4 gene in macrophage or microglia cells alters the inflammatory process to facilitate tissue regeneration [23]. Activation of EphB2-mediated forward signaling stimulates mouse embryonic palate cell proliferation and skull development [18]. Accordingly, these studies indicate that the role of ephrin/Eph bidirectional signaling is complex and dependent on the target tissue, cell-types and developmental stage of bone growth. In our studies, we show that the newly formed bone cells express high levels of ephrin B1 and its cognate EphB2 receptor. We also found, by Western blot analyses, that osteoclast precursors express high levels of the EphA4 receptor during osteoclast differentiation. Thus, we assume that both of the ephrin ligands and the ephrin receptors can function as mediators of bone formation. The interaction of ephrin B1 present on osteoblasts with EphB2 receptors expressed on osteoblasts or osteoprogenitor cells may initiate EphB2 mediated forward signaling to stimulate progenitor cell proliferation, and ephrin B1 mediated reverse signaling to promote osteoblast differentiation. Using the same rationale, the contact of osteoblast with osteoclast can activate ephrin B1 reverse signaling to inhibit osteoclast formation, but also to promote osteoblast differentiation and/or proliferation via ligand-mediated reverse signaling and receptor-mediated forward signaling, respectively. It will be interesting to investigate whether interaction of ephrin B1 with EphB2 receptor in osteoblasts alters RANKL production and osteoclast formation in an osteoblast/osteoclast co-culture system in future.

In our *in vitro* osteoclast differentiation assays, we were surprised to find that the basal level of RANKL-induced osteoclast formation was much lower in the precursors derived from ephrin B1 Tg mice than the cells from WT control littermates in the absence of clustered EphB2-Fc stimulation. It should be noted that we used osteoclast precursors derived from mouse spleens to eliminate potential BMS cell contamination for *in vitro* osteoclast differentiation experiments. One potential explanation why ephrin B1 Tg precursors form fewer osteoclasts *in vitro* is because osteoclasts also express high levels of EphA4, and that endogenous EphA4 interacts with ephrin B1 to activate ephrin B1 mediated reverse signaling. Further studies are needed to find out if EphA4 and/or other Eph receptors interact with ephrin B1 in osteoclast precursors and, thereby, contribute to the observed changes in osteoclast formation and function in Col3.6-Tg^*efnb1*^ mice.

We demonstrate that ephrin B1 expression in the loaded tibia of the Tg mice is much higher than the loaded ones of WT mice. There are a few reasons for the increase in ephrin B1 expression in the loaded bone of the transgenic mice. 1) Osteoblasts and osteoclasts in Tg mice express much higher levels of ephrin B1 than the bone cells of WT mice. 2) Mechanical stress stimulates Col3.6 promoter activity in osteoblasts, as mechanical loading can induce endogenous collagen 1A2 expression in bone. 3) There are more osteoblasts formed or recruited to the loading sites, thus the transgenic gene is expressed more in the loaded area.

It has been previously reported that an interaction between ephrin B2 produced by osteoclasts and EphB4 expressed in osteoblasts contributes to bone homeostasis [8]. In contrast to this report, we failed to detect either ephrin B2 protein using Western immunoblot analyses in osteoclasts during *in vitro* precursor differentiation or EphB4 receptor by immunohistochemisty in TRAP positive osteoclasts *in vivo*. In agreement with our findings, Benson et al. [9] have reported that ephrin B2 protein was undetectable on the periosteal, endosteal, or trabecular bone surface in the adult skull or long bones, and EphB4 expression was not observed in the adult skull [9]. One potential explanation for the inconsistent results is that the bone samples we used were from late bone growth developmental stages or adult bones, and the amount of total cellular protein (30 µg) we used was not sufficient to detect low levels of endogenous ephrin B2 by Western blot with low affinity antibodies. In agreement with Takyar’s recent observation [26], our studies suggest that ephrin B2-mediated reverse signaling may not play an important role in regulating osteoclast formation. Since we observed increased expression of EphB2 in the loaded bones as compared to unloaded bones in WT mice, a finding that is consistent with our earlier report [19], we speculate that a EphB2-ephrin B1 interaction may be involved in mechanical loading-induced bone formation in Col3.6-Tg^*efnb1*^ mice. Accordingly, there are published reports implicating a role for an ephrin B1-EphB2 interaction in regulating regeneration of other tissues. In a mouse ulcer model, EphB2 expression was upregulated in the regenerating epithelium and expanded into the isthmus while ephrin-B1 expression was in pit cells and proliferating cells of the isthmus, where stem cells are located and EphB2-ephrin B1 bidirectional signaling is activated [27]. Stimulation of EphB2 signaling by ephrin B1 controls cell positioning and tissue architecture in the normal and regenerating gastric epithelium [27]. It has also been found that intestinal stem cells express high levels of EphB2 [28] which is required for tissue repair by regulating proliferation and migration of these stem cells. The issue of whether EphB2 and/or other Eph receptors expressed in other cells such as endothelial cells and osteocytes interact with ephrin B1 and contribute to mechanical loading-induced bone formation under normal physiological conditions will be investigated in future studies.

## Materials and Methods

### Recombinant proteins and antibodies

Recombinant proteins of control Fc and EphB2-Fc, anti-human IgG, and anti-β-actin were purchased from Sigma (St. Louis, CO). Recombinant proteins M-CSF, RANKL, and anti-ephrin B1, anti-ephrin B2 and anti-EphB4 antibodies were purchased from R & D Systems (Minneapolis, MN). Anti-osteocalcin and anti-osterix antibodies were purchased from Abcam (Cambridge, MA). Anti-EphA4 antibody was purchased from Cell Signaling Technology (Boston, MA).

### Mice

To generate ephrin B1 Tg mice, a full-length cDNA of mouse ephrin B1 was cloned into a Tg vector which was modified from pWhere (Invivogen, San Diego, CA) and ColCAT3.6 (provided by Dr. David Rowe) as shown in Figure 1A. Gel purified DNA corresponding to the ephrin B1 expression cassette was microinjected into fertilized C57BL/6J mouse ova at the Transgenic Core Facility of the University of California, Irvine. Genotyping was carried out using polymerase chain reaction (PCR) with specific primers to the rCol1A1 promoter region and the transgene coding region. The positive founders were bred with C57BL/6J mice to generate F1 Tg and control mice for each line for subsequent characterization of transgene expression levels and skeletal phenotypes. Mice were housed at the Jerry L. Pettis Memorial VA Medical Center Veterinary Medical Unit (Loma Linda, CA) under standard approved laboratory conditions. All the procedures were performed with the approval of the Institutional Animal Care and Use Committees of the Jerry L Pettis Memorial VA Medical Center. Mice were anesthetized with approved anesthetics (Isoflurane, ketamine/xylazine) prior to procedures. For euthanasia, animals were exposed to CO2 prior to cervical dislocation.

### Mechanical loading

The mice were externally loaded *in vivo* using a four-point bending device as described previously [29]. Because the tibia size was not different between WT and Tg, the calculated strain for the applied loads was not different for the two genotypes. We chose the four point bending method of loading since this regiment caused a robust increase in periosteal bone response at the mid-diaphyseal region of bone where the load was applied. We have characterized the bone formation response to four point-bending extensively in previous studies and demonstrated that the bone formation response was due to mechanical strain and not due to periosteal pressure [30,31]. Briefly, the right tibias of the mice were loaded at 9N, 2Hz for 36 cycles per day and the left tibias of the same mice were used as unloaded controls for 2 weeks with a day break. Twenty-four hours after 2 weeks of stimulation, the mice were euthanized. Tibias were collected, fixed, and skeletal changes were measured by micro-CT.

### Evaluation of bone phenotypes

Micro-architecture of the femurs isolated from 8 week old mice and the tibias from 12 week old mice were assessed by micro-CT (viva CT40, Scanco Medical AG, Switzerland) as described previously [5,32]. The femurs and tibias were fixed in 10% formalin overnight, washed with PBS and immersed in PBS to prevent them from drying. The bone was scanned by X-ray at 55 kVp volts for trabecular bone and mechanical-loading induced new bone at a resolution of 10.5 µm/slice. We use the scout view of the whole bone to measure the bone length. The analyzed regions were 1) in trabecular bone from the distal femur between 0.315 mm and 1.365 mm (second spongiosa) above the distal femur growth plate, 2) in cortical bone of the femur, 0.525 mm long on each side of the center (total 1.05 mm, 100 slides). Samples were taken far away from the growth plate to exclude the primary spongiosa [33]. In mechanical loaded tibia, a 1.05 mm sampling site that was 5.5 mm from proximal tibia growth plate was used for measurement of newly formed cortical bone. Parameters such as bone volume (BV, mm^3^), bone volume fraction (BV/TV, %), trabecular number (Tb.N, mm-^1^), trabecular thickness (Tb.Th, μm) and trabecular space (Tb. Sp, μm) were evaluated [34]. The bones analyzed were adjusted for length so that the regions of interest chosen for cortical and trabecular bone parameter analysis were anatomically the same if there was a difference in bone length between Col3.6-Tg^*efnb1*^ mice and control littermates.

### Dynamic calcein labeling and histomorphometry

Ten week old mice were injected intraperitoneally with calcein eight days (20 mg/kg) and two days prior to the expected day of euthanization in order to label the mineralizing bone surface. Mouse femurs were fixed in 4% paraformaldehyde overnight. The bones were washed, dehydrated, and embedded in methyl methacrylate resin for sectioning. Longitudinal sections of comparable anatomic position of the femurs were analyzed by fluorescence microscopy. For analysis of trabecular bone formation parameters, distal metaphysis between 0.3 mm and 1.4 mm (e.g. second spongiosa) above the distal femur growth plate of left femurs were used as a sampling site. The mineral MAR and BFR/BS were calculated as described previously [35]. For evaluation of bone resorption parameters, the right femurs were partially demineralized, embedded in glycomethacrylate and cut into sections. Seven microscopic fields per bone section that covered all trabecular bone at the second spongiosa of the distal metaphysic femur were used for TRAP surface measurements. Cortical bone and trabecular bone that was adjacent to the growth plate (primary spongiosa) were excluded since active remodeling is taking place at this place. The post coupling method with Napthol-AS-BI phosphate as the substrate and diazotized pararosaniline as the coupling reagent was used for TRAP staining. Two middle longitudinal sections per animal were stained and counted. The trabecular surface and the TRAP labeled trabecular surface were measured in a blinded fashion with OsteoMeasure software (Osteometrics, Inc, Decatur, GA) by our histomorphological core facility [36,37].

### Mineralized nodule formation

BMS cells were isolated from the femurs and the tibias of 4 week old Tg mice and WT control mice as reported previously [38]. Cells were maintained in α-MEM supplemented with 10% fetal bovine serum (FBS), penicillin (100 units/ml), and streptomycin (100 μg/ml) at 37^o^C in 5% CO_2_. To induce differentiation, BMS cells were incubated with α-MEM containing 10% calf serum (CS), β-glycerophosphate (10 mM), and ascorbic acid (50 µg/ml) for 24 days. Nodules were stained for mineralized matrix using alizarin red and mineralized area was quantified as previously described [38].

### *In vitro* osteoclast formation

Primary osteoclast precursors were isolated from the spleen or bone marrow of long bones (femur and tibia) of 5-week old Tg mice and WT littermates as described previously [39]. The isolated precursors were maintained in α-MEM supplemented with 10% fetal bovine serum (FBS), penicillin (100 units/ml), streptomycin (100 μg/ml), and macrophage colony stimulating factor (M-CSF) (20 ng/ml) at 37^o^C in 5% CO_2_ for 2 days to stimulate monocyte proliferation. To induce osteoclast differentiation, trypsinized precursors were seeded into 48-well plates (5000 cells/well) and incubated with M-CSF (20 ng/ml) and RANKL (30 ng/ml). The medium was changed every 2 days. Osteoclastogenesis was evaluated by counting TRAP stained MNCs with more than three nuclei (Sigma Aldrich, St. Louis, MO).

### RNA extraction and quantitative PCR

RNA was extracted from differentiated BMS cells or primary osteoclast cultures as described previously [32]. An aliquot of RNA (2 µg) was reverse-transcribed with an oligo(dT)_12-18_ primer into cDNA in a 20 µl reaction volume. The real time PCR reaction contained 0.5 µl of template cDNA, 1x SYBR GREEN master mix (ABI), and 100 nM of specific forward and reverse primers in a 25 μl reaction volume. Primers for peptidyl prolyl isomerase A (PPIA) was used to normalize the expression data for the genes of interest. The primer sequences used for real-time PCR are listed in Table 1.

**Table 1:** Primer sequences used for real time PCR.

	Forward primer sequence	Reverse primer sequence
PPIA	5’-CCATGGCAAATGCTGGACCA-3’	5’-TCCTGGACCCAAAACGCTCC-3’
Ephrin B1	5’-TGCAACAAGCCACACCAGGA-3’	5’-CGACGGCTGCGAACAATGCT-3’
Ephrin B2	5’-AGCCCTAACCTCTGGGGTCT-3’	5’-GCCATCGGTGCTAGAACCTG-3’
Ephrin B3	5’-AGCACTGTGGACATGATGGACTCT-3’	5’-ACTGCGACTTCCTGTTCTGGATGA-3’
Eph A4	5’-TGATCAAAGCCATCGAGGAAG-3’	5’-TGTCCTCTTCAGGCTGTTGGG-3’
EphB1	5’-ACCATGAGGAGCATCACCTTGTCA-3’	5’-TAGCCCATCGATACGTGCTGTGTT-3’
EphB2	5-TCCTCATCGCTGTGGTCGTC -3’	5’- GGATGACATTGGGGTGGTCG -3’
EphB3	5’-AGTTCGCCAAGGAGATCGATGTGT-3’	5’-TCAGCGTCTTGATAGCCACGAACA-3’
EphB4	5'-CATCAAGGTGGACACAGTGG-3'	5'-TCAAGTTCGTGATCAGCCAG-3'

### Western blot and immunohistochemistry analyses

Western blot analysis was carried out as reported [5,38]. Differentiated BMS cells, primary carvarial osteoblasts and osteoclasts were lysed, and 30 μg of total cellular protein was separated by 8% SDS-PAGE under reducing conditions for Western blot analyses with specific antibodies against ephrin B1, osterix, and β-actin as described previously. Immunohistochemistry was performed using a rat or rabbit immunohistochemistry kit (Vector Laboratories, Burlingame, CA) according to the manufacturer’s instructions. Briefly, tissue sections were de-paraffinized in histochoice clearing agent, rehydrated in a graded series of ethanol and tap water, and treated with 3% H_2_O_2_ for 30 minutes to inactivate endogenous peroxidase activity. The sections were then rinsed thoroughly with PBS (pH 7.4) and heated for 20 minutes at 90 ^0^C in sodium citrate citric acid buffer (pH2.5) for epitope recovery. The sections were pretreated with a blocking solution containing normal goat serum for 20 minutes, and then incubated with primary antibodies at a 1:200 dilution. We have previously confirmed the specificity of these primary antibodies by Western blot analyses. These ephrin B1, B2 and EphB2, B4 antibodies did not cross-react with unintended peptides [5]. Negative control sections were incubated with normal rat IgG or rabbit IgG. After an overnight incubation at 4 ^0^C, the sections were rinsed with PBS, and incubated with biotinylated anti-rat or anti-rabbit secondary antibodies for 30 minutes at room temperature. The sections were then washed in PBS, incubated with VECTASTAIN Elite ABC Reagent for 30 minutes, rinsed again with PBS, and incubated with the Vector VIP substrate until the desired purple stain developed. Immunohistochemistry images were converted to an 8-bit format, and 20 purple stained cells from the areas of newly formed bone were randomly selected to measure the stain intensity with ImageJ software (National Institute of Health). After subtracting the background, the averaged intensity means were used for statistic analysis.

### Statistical Analysis

Data are presented as mean ± standard error of means (SEM) from 6–10 mice from each group. Significant difference was determined as P ≤ 0.05 or P ≤ 0.01. All experiments were reproduced at least twice. Data were analyzed by Student’s t-test or two-way ANOVA.
